# Blood Transcriptome Response to Environmental Metal Exposure Reveals Potential Biological Processes Related to Alzheimer's Disease

**DOI:** 10.3389/fpubh.2020.557587

**Published:** 2020-10-21

**Authors:** Julian Krauskopf, Ingvar A. Bergdahl, Anders Johansson, Domenico Palli, Thomas Lundh, Soterios A. Kyrtopoulos, Theo M. de Kok, Jos C. Kleinjans

**Affiliations:** ^1^Department of Toxicogenomics, Maastricht University, Maastricht, Netherlands; ^2^Section of Sustainable Health, Department of Public Health and Clinical Medicine, Umeå University, Umeå, Sweden; ^3^Odontology, Umeå University, Umeå, Sweden; ^4^Cancer Risk Factors and Life-Style Epidemiology Unit, Institute for Cancer Research, Prevention and Clinical Network - ISPRO, Florence, Italy; ^5^Division of Occupational and Environmental Medicine, Lund University Hospital, Lund, Sweden; ^6^Institute of Chemical Biology, National Hellenic Research Foundation, Athens, Greece

**Keywords:** Alzheimer's disease, metals, gene expression, transcriptomics, microarray, APOE

## Abstract

Alzheimer's disease (AD) is a neurodegenerative disease which is manifested by a progressive and irreversible decline of cognition, memory loss, a shortened attention span, and changes in personality. Aging and genetic pre-dispositions, particularly the presence of a specific form of apolipoprotein E (*APOE*), are main risk factors of sporadic AD; however, a large body of evidence has shown that multiple environmental factors, including exposure to toxic metals, increase the risk for late onset AD. Lead (Pb) and cadmium (Cd) are ubiquitous toxic metals with a wide range of applications resulting in global distribution in the environment and exposure of all living organisms on earth. In addition to being classified as carcinogenic (Cd) and possibly carcinogenic (Pb) to humans by the International Agency for Research on Cancer, both compounds disrupt metal homeostasis and can cause toxic responses at the cellular and organismal levels. Pb toxicity targets the central nervous system and evidence for that has emerged also for Cd. Recent epidemiological studies show that both metals possibly are etiological factors of multiple neurodegenerative diseases, including Alzheimer's disease (AD). To further explore the association between metal exposure and AD risk we applied whole transcriptome gene expression analysis in peripheral blood leukocytes (PBLs) from 632 subjects of the general population, taken from the EnviroGenomarkers project. We used linear mixed effect models to associate metal exposure to gene expression after adjustment for gender, age, BMI, smoking, and alcohol consumption. For Pb exposure only few associations were identified, including a downregulation of the human eukaryotic translation initiation factor 5 (*eIF5*). In contrast, Cd exposure, particularly in males, revealed a much stronger transcriptomic response, featuring multiple pathways related to pathomolecular mechanisms of AD, such as endocytosis, neutrophil degranulation, and Interleukin−7 signaling. A gender stratified analysis revealed that the Cd responses were male-specific and included a downregulation of the *APOE* gene in men. This exploratory study revealed novel hypothetical findings which might contribute to the understanding of the neurotoxic effects of chronic Pb and Cd exposure and possibly improve our knowledge on the molecular mechanisms linking metal exposure to AD risk.

## Introduction

Alzheimer's disease (AD) is a progressive neurodegenerative disease and the most common form of dementia. Currently, close to 50 million people are afflicted with dementia across the globe, and this number is estimated to triple by 2050 (1). AD is manifested in a progressive and irreversible decline of cognition, memory loss, a shortened attention span and changes in personality (2).

Despite decades of research, the etiopathology of AD remains largely unknown, and no effective treatment is currently available. Pathological hallmarks include a degenerative process of damaging cholinergic neurons across brain regions that serve important functional roles in conscious awareness, attention, and memory; extracellular aggregates of amyloid beta (Aβ) peptides resulting in disrupted cell functioning; intracellular accumulation of hyperphosphorylated tau, causing synaptic dysfunction, along with glial activation, and inflammation (2, 3).

A rare form of AD is early-onset, or familial AD, which accounts for only 2–10% of total AD cases and occurs at ages below 65. Genome-wide association studies have revealed three high-penetrance mutations to cause autosomal dominant AD: Amyloid precursor protein (*APP*), Presenilin 1 (*PSEN1*), and Presenilin 2 (*PSEN2*) (4). However, the vast majority of patients suffer from late onset, or sporadic AD, with clinical symptoms only occurring at an age above 65 years. Sporadic AD is a multifactorial disease with aging and genetic pre-dispositions, particularly the presence of the apolipoprotein E ε4 (APOE ε4) allele, being the main risk factors. Moreover, epidemiological studies have also identified multiple environmental risk factors, including exposure to toxic metals, as drivers in late onset AD (4–7).

Humans are exposed to toxic metals through inhalation of contaminated air, dermal absorption of metals contained in soil, and ingestion of contaminated water and food (7). Lead (Pb) and Cadmium (Cd) are naturally occurring metals that are widely used in e.g., industrial and domestic applications. They also occur in some agricultural applications. This has resulted in a global distribution in the environment. Even at low levels of exposure, Pb, and Cd can induce multiple organ damage, and therefore present a major human health threat. Cd has been classified as carcinogenic and Pb as possibly carcinogenic to humans by the International Agency for Research on Cancer (8). Next to the carcinogenic effects, a large body of evidence has emerged that Pb and Cd toxicity also target the central nervous system (CNS), causing neurodegenerative effects (9), and increasing evidence has demonstrated that both Pb and Cd exposure are possible etiological factors for sporadic AD. Population-based research has shown that Pb, and possibly also Cd, exposure results in AD-like pathology such as memory loss and deficits in intelligence, attention, language, and emotion (10, 11).

Once in the blood, Pb is distributed throughout the body, thereby making it available to other tissues (12). In the brain vessels Pb substitutes for calcium ions, and can therefore rapidly cross the blood-brain barrier (13). Brain Pb levels alter neuronal differentiation and cause severe damage to the brain (14). Even low Pb levels have been shown to cause neurotransmitter alterations, possibly resulting in malfunctioning of the GABAergic, dopaminergic, and cholinergic systems (15). Intracellularly, Pb replaces calcium, and other essential metals, and disrupts the corresponding biometal-dependent mechanisms (12). Pb exposure was associated to increased levels of Aβ peptides in animal and cell culture studies (16, 17). In rodents, increased Pb levels were also associated to elevated expression of APP as well as increased production of Aβ peptides (18). A case study has reported that Pb poisoning in childhood has led to neurofibrillary tangles in the brain (19). A study on 55 young adults who had participated as newborns in a prospective cohort study showed inverse correlations of umbilical cord Pb concentration and expression of potential AD biomarkers e.g., ADAM9, RTN4, and LRPAP1 genes, hence demonstrating that early-life Pb exposure influences biological processes involved in AD pathogenesis (20).

Chronic Cd exposure is associated with multiple health outcomes including hypertension, kidney dysfunction, and also neurological diseases (12). Cd has been shown to cross the blood-brain barrier, and has even been shown to alter its permeability (21). The mechanisms underlying Cd neurotoxicity are not completely understood, however, they include oxidative damage, inflammation, neuronal apoptosis, and possibly interaction with other metals such as cobalt and zinc (12, 22). Other studies have shown that Cd also interacts with neurotransmitters, e.g., causing a decrease in exocytotic release of glutamate (23), possibly by blocking the influx of Ca2+ through membrane channels (24).

The potential role of Cd in sporadic AD arises from the presence of significantly higher Cd concentrations in brain tissue of AD patients as compared to age-matched control subjects (25). *In vitro* experiments have illustrated that Cd potentially causes self-aggregation of the tau peptides (26), and may directly interact with Aβ protein (27).

Evidently, humans are not exposed to single metals but rather to mixtures, which makes it even more complicated to identify molecular mechanisms triggered by individual compounds. Several studies have shown that chemicals, including heavy metals, can enhance the effect of other chemicals, so that as a mixture they can exert a larger effect (28). This synergistic effect for Pb and Cd has been shown to occur in model organisms (29). In addition it has been suggested that high Pb levels affect mental and psychomotor development in children exposed to high prenatal Cd levels (30).

Together, these studies suggest potential implications of Pb and Cd in the pathomolecular mechanisms which might increase sporadic AD risk, although further research is needed to better understand the neurotoxic mechanisms of chronic low-level Pb and Cd exposure, as well as its potential role in the etiopathology of AD. In the present study we assume that Pb and Cd induce responses in blood cells that are similar to responses in the CNS. Previous studies have shown that differentially expressed genes in the brain and blood revealed a significant overlap of gene expression patterns (31, 32). Therefore, we have applied whole transcriptome gene expression analysis in blood cells from 632 apparently healthy subjects chronically exposed to Pb and Cd from the EnviroGenomarkers project (http://www.envirogenomarkers.net).

## Materials and Methods

### Selection of the Population

To investigate the effect of metal exposure on disease risk we performed a global transcriptome analysis in archived peripheral blood leukocytes (PBLs) samples of 632 and exposure/health data were derived from -at that time- generally healthy subjects from the EnviroGenomarkers Project. The EnviroGenomarkers project was designed as a nested case-control study, including controls and “future disease” subjects developing either lymphoma or breast cancer after a minimum 2 years from sample collection. The project is based on two prospective cohort studies that have previously been described: The Northern Sweden Health and Disease Study (NSHDS) (33), in which its subcohort the Vasterbotten Intervention Programme (*n* = 406) (34) was used, and EPIC-Italy (*n* = 226) (35). For the present study we analyzed blood samples from 632 participants, including 393 females and 239 males (Table 1). Smoking status was assessed at the time of sampling. The EnviroGenomarkers project was approved by the Regional Ethical Review Board in Umea and the Florence Health Unit Local Ethical Committee and all methods were carried out in accordance with the approved guidelines. All participants gave written informed consent.

**Table 1:** Population characteristics. Smoking status was self-reported at the time of blood sampling.

**Population**		**Age**		**BMI**		**Smoking**		
		**Range**	**Mean**	**Range**	**Mean**	**current**	**non**	**never**
Total	632	30, 75	52	19, 40	26	144	140	348
Males	239	30, 75	53	19, 49	27	44	76	119
Females	393	30, 65	51	19,39	26	100	64	229
EPIC	226	37, 75	53	19, 36	26	58	60	108
NSHD	406	30, 61	52	19, 40	26	86	80	240

### Exposure Assessment

Erythrocyte concentrations of Pb and Cd were determined by means of inductively coupled plasma–mass spectrometry (ICP–MS) (36) at Lund University Hospital, Lund, Sweden, for both the EPIC–Italy and NSHDS samples. The limit of detection, calculated as three times the standard deviation of a blank, was 0.03 μg/L for cadmium and 0.09 μg/L for lead in EPIC–Italy and NSHDS samples, as described earlier (37). In addition to the numerical variable for each metal we calculated a ZScore for each subject as a representative of the internal exposure to the mixture of both metals. This ZScore is centered on the population mean and therefore negative values correspond to the raw scores below the population mean, while positive values correspond to the raw scores above the population mean. The ZScore was defined as the mean of the ZScores for each metal: ZScore = (X–μ)/σ (where X represents the value of the subject, μ the mean and σ the standard deviation of the population) (38–40).

### Analytical Procedures

PBLs for RNA extraction were taken at the same time as samples for measurements of metal concentrations. Total RNA was extracted from PBLs with the RiboPure™ Blood kit (Ambion, Austin, TX, United States). Genome-wide analysis of gene expression (Agilent 4 × 44K human whole genome microarray platform) and the corresponding data quality assessment and preprocessing, were conducted as described previously (41, 42). Briefly: RNA samples were reverse transcribed into cDNA and labeled with cyanine 3 prior to hybridization. Subsequently, slides were washed and scanned using the Agilent Technologies G2565CA DNA Microarray Scanner. Quality control was established by visual evaluation of the scan images before and after within- and between-array normalization (using LOESS and A-quantile, respectively) (42). Probes with <75% of the maximum possible number of pixels were left out. Imputation of the missing values was done using k nearest neighbors approach (41). Resulting signal intensities were log2 transformed before proceeding.

### Statistical Data Analysis

Linear mixed models (LMM) were used to identify associations between the gene expressions and the individual Pb and Cd levels as well as the cumulative ZScore. The analyses were performed using the R package “lme4,” correcting for the potential confounders gender, age, BMI, smoking and alcohol intake. Furthermore, the technical variations introduced by the different batches of RNA isolation, RNA labeling and hybridization were added as random effects. Covariates included in the model were selected by the following method: Using the R package “lmer/lm” we performed a model m0 explaining the variation in the transcriptome by exposure, and a model m1 explaining the variation in the transcriptome by exposure and controlling for a single covariate. Both models were subjected to an anova test and resulting *p*-values were plotted in a pqq plot. If the distribution of *p*-values from the respective anova deviated significantly from the normal distribution a covariate was included in the final model (Supplementary Figure 1). Scaled, numerical Pb, Cd and the cumulative ZScore were defined as the respective variables of interest. Resulting *p*-values were controlled for by the False Discovery Rate (FDR) set at 5 and 20%. Reactome and KEGG pathway analyses were performed using the R package “enrichPlot.” Furthermore, we have used Cytoscape (version 3.8.0) and the Reactome FI package to perform a network analysis, pathway enrichment analysis and gene set enrichment analysis (GSEA). The GeneScore used for the GSEA and network analysis was calculated by multiplying the β-coefficient and –log10 of the corresponding *p*-value. A full KEGG pathway analysis was done using the web interface of ConsensusPathDB. To test the stability of the model we performed modified k-means cross validation. Briefly: 10% of the subjects were randomly left out at a time; LMM was performed on the remaining 90% subjects. This step was repeated for 100 times. At the end, we compared all resulting 100 coefficient vectors with the coefficient vector of the full dataset set including all subjects.

## Results

### Population and Exposure

To investigate the effect of metal exposure on disease risk we performed a global transcriptome analysis in PBL samples of 632 subjects from the EnviroGenomarkers study population of 393 females and 239 males (Table 1).

Next to the internal exposure to the individual pollutants Pb and Cd we also attempted to investigate the potential synergistic effect using a cumulative ZScore of both metals. Across the cohorts we found significantly higher exposure levels in EPIC-Italy for ZScore and Pb when compared to NHDS. For cadmium no significant differences were observed (Table 2). Males showed significantly higher levels of Pb exposure, while females had higher Cd levels. The ZScore, however, did not show a significant difference between genders (Table 3).

**Table 2:** Exposure metrics in the two cohortd NSHDS and EPIC-Italy.

	**EPIC**			**NSHD**			***P*-value**
	**Range**	**Mean**	**SD**	**Range**	**Mean**	**SD**	
Zscore	−0.61, 4.85	0.33	0.33	−0.89, 5.57	−0.18	0.71	**<2.2e-16**
Lead [μg/L]	29.3, 401	102	50.9	12.4,672	52.2	46.1	**<2.2e-16**
Cadmium [μg/L]	0.18, 3.61	0.85	0.61	0.1, 5.22	0.78	0.087	0.2384

*The table shows the range, mean and SD for erythrocyte Pb and Cd concentrations and the cumulative ZScore in the two cohorts. P-values originated from a paired t-test. P-values show the level of significance between the two cohorts. P < 0.05 are marked in bold*.

**Table 3:** Exposure metrics in males and females.

	**Males**			**Females**			***P*-value**
	**Range**	**Mean**	**SD**	**Range**	**Mean**	**SD**	
ZScore	−0.86, 5.57	0.02	0.85	−0.89, 4.85	−0.01	0.66	0.6788
Lead [μg/L]	15.4, 672	82	68.7	12.4, 401	62.7	40	**9.457e-05**
Cadmium [μg/L]	0.1, 5.22	0.66	0.79	0.16, 4,32	0.9	0.78	**0.0001796**

*The table shows the range, mean, and SD for erythrocyte Pb and Cd concentrations and the cumulative ZScore in males and females. P-values originated from a paired t-test. P-values show the level of significance between the two cohorts. P < 0.05 are marked in bold*.

### Transcriptome-Wide Metal Exposure Associations

**Figure 1:**
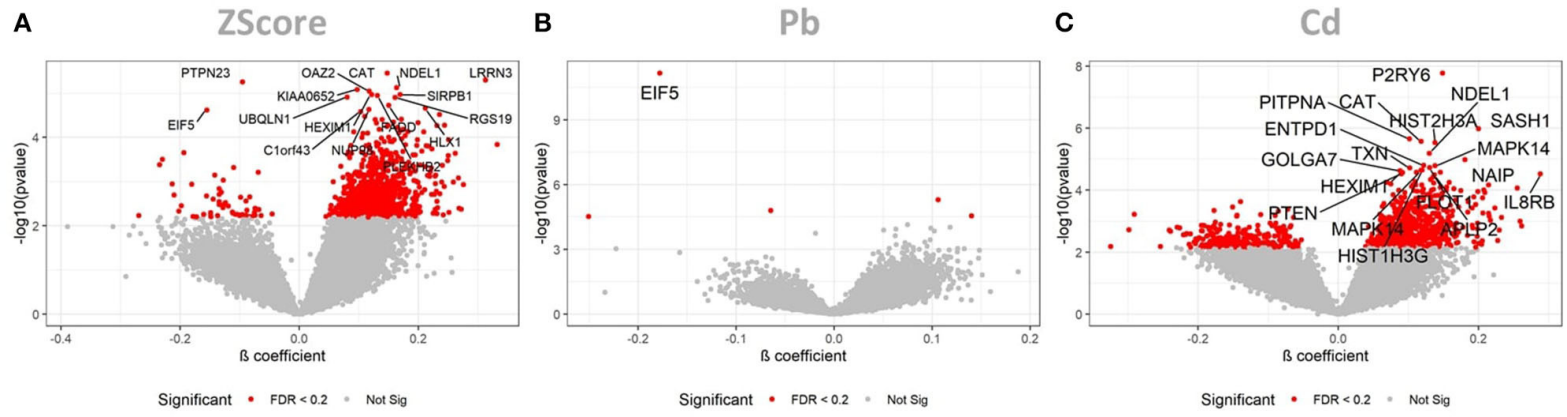
LMM results for the full dataset. These volcano plots show the resulting *p*-values of the LMM when associating the AgIDs to the cumulative metal ZScore **(A)**, individual Pb **(B)** and Cd **(C)** exposure. The red color indicates transcripts significant with a FDR <20%; labeled transcripts remained significant at FDR < 0.05.

The LMM identified the cumulative ZScore of metal exposure to be significantly associated to 16 Agilent Probe IDs (AgIDs) when controlling the FDR at 5 percent, and 954 AgIDs when controlling the FDR at 20 percent. These corresponded to 16 (14↑, 2↓) and 767 (734↑, 33↓) transcripts, respectively (Figure 1A). The individual pollutant Pb was significantly associated with only 1 AgID when controlling the FDR at 5 percent, and 5 AgIDs when controlling the FDR at 20 percent. These corresponded to 1 (↓) and 4 (2↑, 2↓) transcripts, respectively (Figure 1B). For Cd the LMM identified 20 AgIDs when controlling the FDR at 5 percent, and 1,039 AgIDs when controlling the FDR at 20 percent. These corresponded to 17 (17↑) and 792 (606↑, 186↓) transcripts, respectively (Figure 1C). The cumulative ZScore showed 3 overlapping transcripts with Pb and 389 with Cd (FDR at 20%). Consequently, 374 transcripts were only identified with the ZScore and are therefore potentially associated to the synergistic effect of Pb and Cd. Since relatively few associations were identified, even taken the low significance threshold of FDR <20%, we hypothesized that gender-differences might have an impact on the analysis. Consequently, we performed a subsequent analysis on the data stratified for males and females.

### Gender-Specific Response

**Figure 2:**
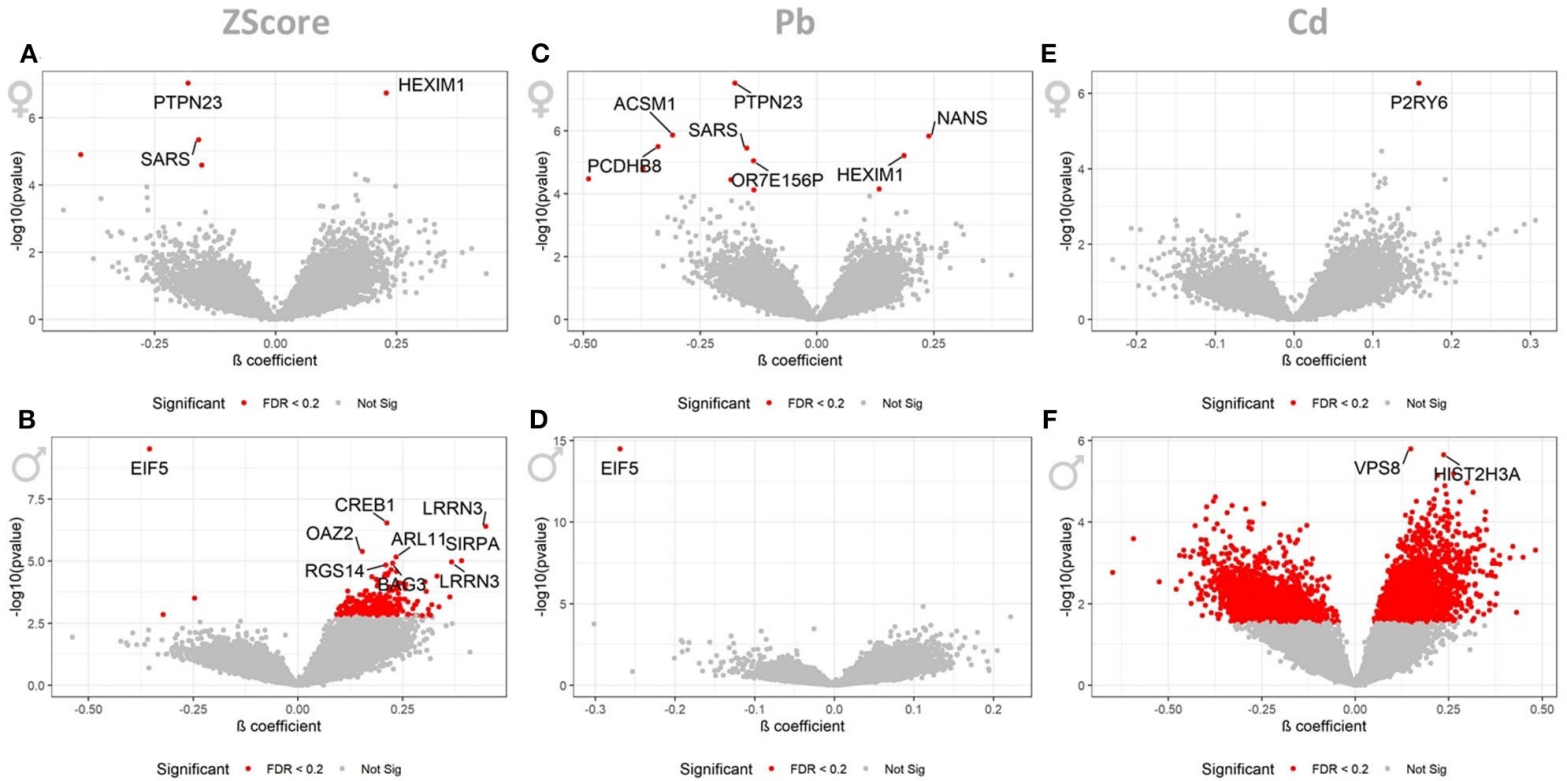
LMM results for the gender stratified dataset. These volcano plots show the resulting *p*-values of the LMM when associating the AgIDs to the cumulative ZScore for females **(A)** and males **(B)**, individual Pb for females **(C)** and males **(D)** and Cd for females **(E)** and males **(F)** exposure. The red color indicates transcripts significant with a FDR <0.2; labeled transcripts remained significant at FDR <0.05.

When stratifying the dataset by gender we observed the following associations: the cumulative ZScore was associated to 3 and 5 AgIDs in the 393 females when controlling the FDR at 5 and 20 percent, respectively. These corresponded to 3 (1↑, 2↓) and 4 (1↑, 3↓) transcripts, respectively (Figure 2A). For the 239 males we identified 9 and 235 associated AgiDs which corresponded to 9 (8↑, 1↓) and 199 (198↑, 1↓) transcripts, respectively (Figure 2B). The individual pollutant Pb was significantly associated with 7 and 12 AgIDs in females when controlling the FDR at 5 and 20 percent. These corresponded to 7 (2↑, 5↓) and 11 (3↑, 8↓) transcripts, respectively (Figure 2C). For males, however, we identified only 1 (1↓) AgID/transcript at both significance thresholds (Figure 2D). For the individual Cd we identified only 1 (1↑) AgID/transcript in females at both significance thresholds (Figure 2E). In contrast, we identified 2 and 4204 AgIDs in males when controlling the FDR at 5 and 20 percent. These corresponded to 2 (2↑ and 2862 (1,662↑, 1,200↓) transcripts, respectively (Figure 2F). The strong male-specific response to Cd exposure, observed at the lower significance threshold of FDR <20%, prompted us to focus the following analysis of potentially perturbed pathways in males only.

### Cross Validation

To validate the stability of the results we performed a modified k-means cross validation (see methods). The cross validation revealed that the results of the cumulative ZScore and Cd showed high stability, while the results of Pb only resulted in moderate stability for full and stratified analysis (Supplementary Figure 1). A stratified LMM analysis for each individual cohort was implemented; however, only few significant associations were found as a consequence of the smaller sample size. Briefly; for the ZScore 27 and 1 AgIDs, and for Pb 3 and 3 AgIDs were identified for EPIC and NHDS, respectively (FDR at 20%). No associations for Cd were identified (FDR at 20%).

To test the impact of a future cancer on our findings we stratified the data to only controls. Despite the small sample size (*n* = 313) we identified 3, 2, and 763 AgIDs to be associated with the ZScore, Pb, and Cd, respectively. Of these findings in controls 2, 1, and 387 AgIDs were overlapping with the associations identified in the full dataset. Stratified analysis for future lymphoma (*n* = 239) and breast cancer (*n* = 80) cases returned no significant associations for Cd and were deemed underpowered.

Since smoking is a major source of cadmium exposure we also stratified our data into never-smokers, non-smokers, and smokers. For never- and non-smokers we did not find any significant association, while for the smokers we only observed 12 positively associated AgIDs (FDR at 20%).

### Perturbed Disease Pathways and Network Analysis

**Figure 3:**
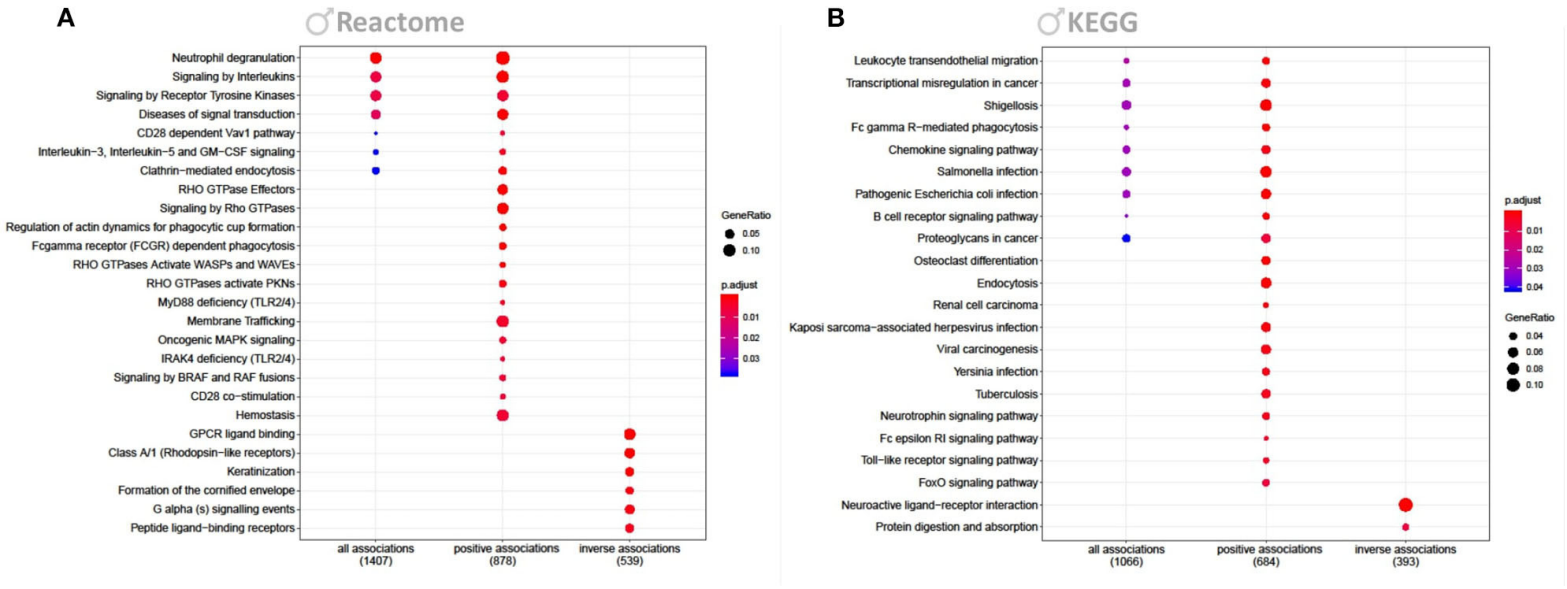
Reactome and KEGG overrepresentation analysis. Figure shows enriched Reactome **(A)** and KEGG **(B)** pathways for genes significantly associated to Cd in males (FDR <0.2). Each figure shows enriched pathways for all, only positive and only inversely associated transcripts. *P*-values of the enrichment analysis have been corrected by FDR and are indicated by color. Bullet sizes represent the gene ratio of transcripts from the associated genes. Numbers in brackets are transcripts that were mapped to the listed pathways.

The strong response to Cd in males prompted us to focus the downstream analysis of perturbed pathways solely on this gender-specific effect. Pathway analysis of all Cd-associated transcripts showed an overrepresentation of genes involved in Reactome pathways such as interleukin signaling, clathrin-mediated endocytosis, and neutrophil degranulation (Figure 3A); KEGG pathways included chemokine signaling (Figure 3B). Figures only include the top 20 significant pathways. Additionally we attached a full list of enriched Reactome and KEGG pathways, and a GSEA (Supplementary Tables 1–3). Interestingly, taken the known accumulation of Cd in kidney, the KEGG pathways analysis revealed a significant enrichment of genes involved in renal cell carcinoma (Figure 3, Supplementary Table 2). When feeding only positively associated transcripts into the analysis we also identified Reactome pathways among others related to hemostasis, rho GTPase, and membrane trafficking (Figure 3A); KEGG pathways also featured such as endocytosis, neurotrophin signaling pathways, and Toll-like receptor signaling (Figure 3B). Inversely associated transcripts identified Reactome pathways such as related to GPCR and Keratinization (Figure 3A); KEGG pathways also featured such as neuroactive ligand-receptor interaction (Figure 3B).

**Figure 4:**
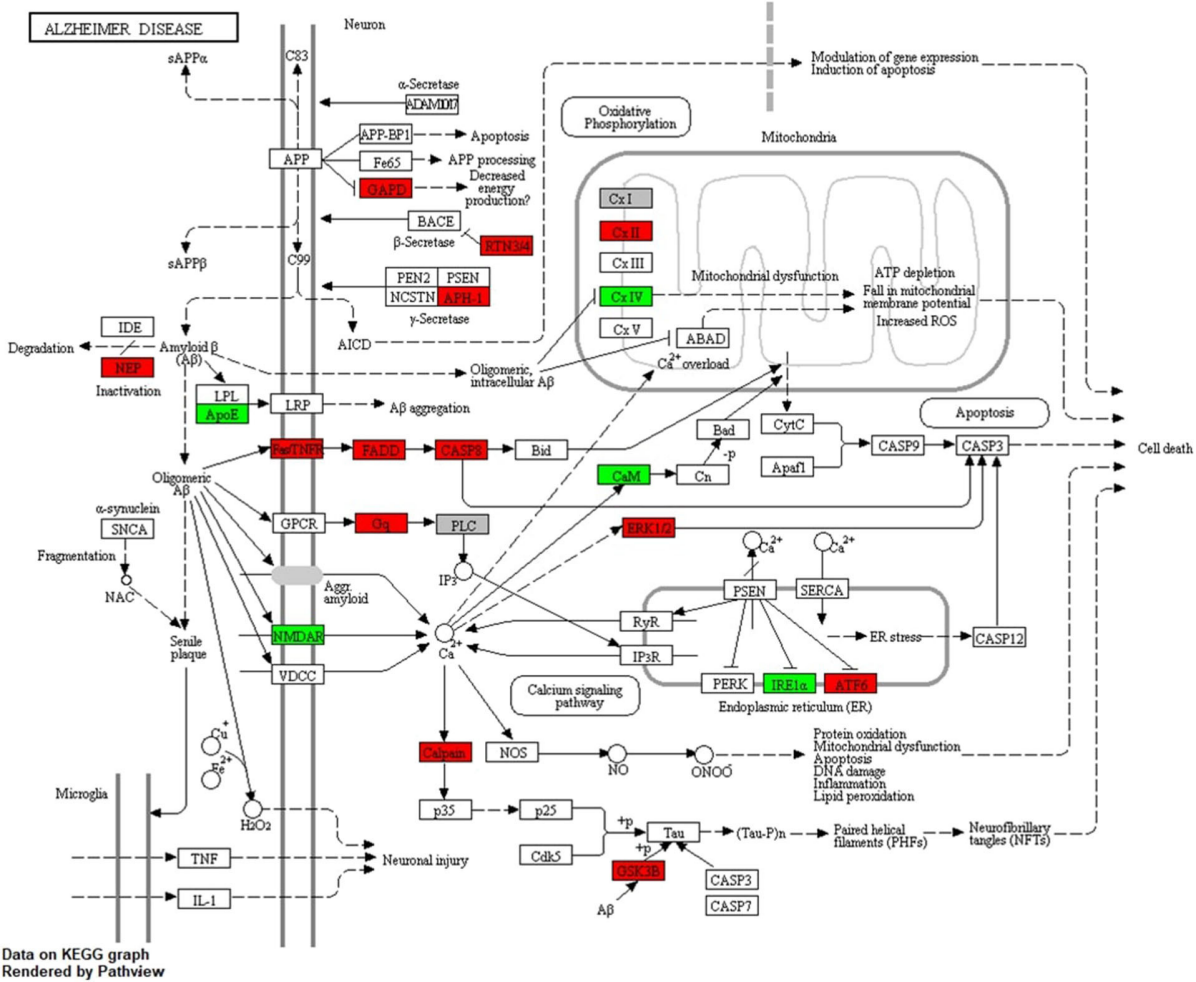
KEGG AD pathway including Cd-associated transcripts in males. The figure shows the LMM results for transcripts associated to Cd exposure in males after mapping onto the AD pathway from KEGG. Red color indicates significant positive and green significant inverse associations with Cd exposure.

The identified response on enriched pathways was not specific to AD, but included AD relevant pathways e.g., inflammatory response and endocytosis. To obtain more specific information on the cellular perturbations as a result of Cd exposure we mapped all Cd-associated transcripts onto the KEGG AD pathway (Figure 4) and identified in total 14 (4↑, 10↓) transcripts to be affected. Hence, we have identified these 14 transcripts to be potentially involved in the molecular modes of action by which Cd exposure influences AD risk in males. We assessed the overlap of the Cd associated transcripts with the reported 470 genes of a recent genetic meta-analysis of diagnosed AD patients (43). We identified 101 genes overlapping with our list of identified genes. A hypergeometric test returned a significant overlap (*p* = 0.02, Supplementary Figure 2A). Furthermore, we also screened for overlap with a recent single cell transcriptomic analysis of AD (44) and identified 100 genes overlapping with our list and 756 genes found in no-pathology vs. pathology in excitatory neurons. The hypergeometric test, however, showed that this was not a significant overlap (p = 0.99, Supplementary Figure 2B). Additionally, we explored the Genotype-Tissue Expression (GTEx) database to see if the identified genes are also expressed in blood. We tested the top 50 Cd associated genes ranked by significance which were also present in the GTEx database and revealed that the expression of these genes in whole blood is largley reassembled in brain and CNS tissue (Supplementary Figure 3).

Additionally, we constructed a protein-protein interaction (PPI) network of the 2,862 Cd associated genes in males. The network was based on PPIs derived from Reactome FIs using Cytoscape. We clustered the data by using the integrated MCL Cluster function to construct networks with a minimum of 40 connected nodes. This resulted in 8 subnetworks (Supplementary Figures 4A–H). We colored the nodes by the previously calculated GeneScore derived from the multiplication of the β-coefficient and –log10(*p*-value). Consequently, red shades indicates a significant positive, a blue shade a significant negative association of the respective transcripts. The networks show that perturbations are centered around multiple hub genes related to cancer such as RAC1, MAPK1, TP53, UBA52 (Supplementary Figures 4A–E), as well as inflammation incl. TYK2, JAK3, and STAT3 (Supplementary Figure 4D).

## Discussion

The present study investigates the effect of metal exposure on molecular mechanisms involved in the etiology of AD by using the transcriptomic response of human PBLs as a surrogate model of the CNS. The whole transcriptome analysis of human subjects exposed to Pb and Cd revealed a pollutant- and gender-specific response.

It remains presently unknown whether single metals or rather, mixtures of environmental factors contribute to AD onset. We aimed to model synergistic effects of Pb and Cd by using a cumulative ZScore; however, since very few associations were found for Pb, and many associations were identified in the ZScore only, we consider these results suggestive and consequently, we have focused on the effect of individual Pb and Cd exposure.

Pb exposure was only associated with 4 (1↑, 3↓) transcripts (e*IF5, PIM1, PTPN23*, and *SLC20A1*), with the downregulation of the human eukaryotic translation initiation factor 5 (*eIF5*) being by far the most significant observation (Figure 1B). During initiation of cytoplasmic translation, *eIF5* forms together with other proteins the 43S preinitiation complex. Translation initiation and elongation plays an important role in learning and memory, neuronal perturbations of translational control have been linked to numerous cognitive disorders (45, 46). Subtle disruptions in translational regulation can have dramatic consequences for survival of neurons, especially in the aging brain (47). Hypothetically, inappropriate reductions of translation initiation, e.g., through a suppression of *eIF5* by chronic Pb exposure, might contribute to the pathogenesis of AD. When stratifying the data for gender we identified a slightly stronger response to Pb in females 11 (3↑, 8↓) (Figure 2C), thereby losing the association to *eIF5*. For males, however, only the inverse association of *eIF5* remained significant (Figure 2D).

The LMM identified 792 (606↑, 186↓) transcripts to be associated with internal Cd exposure. Among the multiple neuroinflammatory genes with increased expression (e.g., *PTEN, MAPK14, CAT, IL8RB, SASH1, TXN*), the most significant association was with increased *P2Y6* expression (Figure 1C). Interestingly, accumulating evidence reveals a prominent role for *P2Y* receptors in AD pathology. Subtle changes in P2Y receptor expression have been shown to influence molecular pathways relevant for AD pathology, including Aβ production and elimination, neuroinflammation, neuronal function, and cerebral blood flow [reviewed in (48, 49)]. A recent study has identified microglia-activated neuroinflammation through increased levels of *P2Y6* in Parkinson disease patients when compared to controls (50).

For females we only observed the previously discussed association of Cd and *P2Y6* (Figure 2E). Remarkably, for males we identified a very strong response to Cd exposure (Figure 2F) consisting of 2,862 (1,662↑, 1,200↓) transcripts. This is the first study showing a gender-specific effect of Cd exposure on relevant AD genes in human. In a very recent study AD mouse models, that expressed either the E4 or E3 alleles of the human APOE gene, were exposed to low-doses of Cd. Particularly male mice were vulnerable to the observed accelerated cognitive impairment induced by Cd (51). In line with the suggested gene-environment interaction between Cd and the *APOE* gene (51), our study identified an inverse association between Cd and APOE transcript (β-coefficient = −0.22; p = 0.0050; FDR = 0.1191) in males only. Multiple studies have reported neuronal degeneration and increased AD risk with increased expression of *APOE* transcript (52). In transgenic models, mouse *APOE* expression appears to increase Aβ deposition (53); however, other studies have also shown that human APOE expression decreases brain Aβ load (54, 55). A study on 114 patients with early and sporadic AD reported 60% decrease in *APOE* expression in lymphocytes from AD cases vs. controls, and also reported an inverse correlation with Aβ. The study concluded that reduced *APOE* expression in human may influence risk and constitute a determinant factor in Aβ loading in AD (54). Consequently, based on our lymphocyte transcriptome analysis, we suggest that Cd-dependent, chronic suppression of *APOE* increases Aβ deposition, suppresses Aβ clearance and/or perturbs other downstream molecular pathways, thus increasing AD risk. Furthermore, we observed an upregulation of the membrane metallo-endopeptidase (*MME*), an enzyme involved in Aβ metabolism (56), as well as *FADD* and *CASP8*, both suspected to mediate Aβ induced neuronal apoptosis (57). Moreover, we identified a perturbed expression of genes associated to decreased energy production and mitochondrial dysfunction with an upregulation of *GAPDH and SDHA*, and a suppression of *COX7A1* (58), upregulation of transcripts related to neuronal cell death such as MAPK1 and GSK3B (59), and multiple deregulated genes involved in the calcium signaling (60) (Figure 4, Table 4).

**Table 4:** LMM results for AD relevant genes identified in males associated to Cd exposure.

**Gene Symbol**	**β-coefficient**	***p*-value**	**FDR**
*ERN1*	−0.23	0.0032	0.1098
*APOE*	−0.22	0.0050	0.1191
*COX7A1*	−0.21	0.0142	0.1561
*CALML4*	−0.21	0.0085	0.1346
*MAPK1*	0.08	0.0244	0.1886
*GAPDH*	0.08	0.0255	0.1927
*GSK3B*	0.09	0.0214	0.1797
*CAPN1*	0.10	0.0057	0.1217
*SDHA*	0.11	0.0111	0.1434
*ATF6*	0.13	0.0036	0.1115
*TNFRSF1A*	0.14	0.0131	0.1491
*GNAQ*	0.14	0.0091	0.1366
*FADD*	0.15	0.0014	0.0914
*CASP8*	0.17	0.0119	0.1469
*MME*	0.31	0.0120	0.1465

*The table shows the LMM results for transcripts associated to Cd exposure in males after mapping to the AD pathway from KEGG*.

We subjected the list of Cd-associated transcripts derived from males to a Reactome and KEGG overrepresentation analysis and identified multiple pathways associated to neuroinflammation (Figure 3). Thereby we identified an upregulation of neutrophil degranulation, the process by which the neutrophils induce inflammation. A large body of publications report on how activated neutrophils in the brain vessels play an important role in the pathogenesis of BBB damage, and subsequent injury of surrounding neurons and AD etiology [reviewed in (61)]. Furthermore, several other inflammatory pathways linked to AD etiology were increased in our data such as hemostasis (62), signaling by interleukins (63), and chemokines (62, 64) as well as TLR signaling (65).

We also identified an overrepresentation of upregulated genes involved in neurotrophin signaling and endocytosis. The signaling endosome hypothesis and the role of neurotrophic factors and endocytosis in AD pathogenesis have been reviewed (66). The upregulation of endocytosis is thought to increase the chances of proteins from the cell surface being packaged into endosomes. As a consequence of this process, increasing amounts of APP are brought into contact with the secretases of the endosomes and increase Aβ production (67). The importance of endocytosis in AD is further supported by the fact that enlarged endosomes are linked to age, and have also been reported in postmortem AD patients (68, 69).

Owing to the design of the EnviroGenoMarkers project the investigated population is below the age to develop AD. Furthermore, no follow up of the participants with respect to AD diagnosis has been made, and subjects were not enrolled in memory performance tests. Therefore, our study can be solely considered an exploratory effort with the aim to identify novel hypothetical association's between heavy metal exposure and molecular pathways which are potentially involved in the etiology of AD. Certainly, these findings have to be investigated in a more dedicated population.

The difference in Pb levels between both cohort and genders is most likely a result from different diets (70). In our statistical model we adjusted for the confounder cohort. All associations were only found when combining both cohorts. Certainly, we cannot exclude that there was any residual confounding; however, the stratified analyses per cohort did not return any significant associations.

The higher Cd levels in females could be a reason for a slightly higher ratio of smokers in the females (males 18% vs. females 25%). Even with the adjustment of smoking in the LMM of Cd exposure, we could not exclude any residual confounding of smoking. Therefore, we have performed a stratified analysis to never-smokers, non-smokers, and smokers. In smokers we observed only 12 positively associated AgIDs, which were also associated with Cd exposure (FDR at 20%). We also assessed the overlap of Cd associations with associations found in a previous study on the EnviroGenomarkers project assessing the influence of smoking on transcriptomics (41). The study identified 351 smoking associated AgIDs, of which only 7 overlapped with the identified Cd associated AgIDs in males. Together, these outcomes indicate that the residual confounding of smoking on our Cd analysis is limited.

To summarize, the data suggest that Pb exposure affects the transcription initiation possibly by neuronal perturbation of the 43S preinitiation complex through downregulation of *eIF5*, while Cd exposure, particularly in males, induces *APOE* suppression, neuroinflammation, and increased endocytosis. These findings possibly contribute to the understanding of the neuronal molecular response of chronic Pb and Cd exposure as well as improve our knowledge on the molecular mechanisms linking metal exposure and AD etiology.

## Data Availability Statement

The datasets generated and analyzed during the current study are not publicly available due to restrictions imposed by Swedish legislation on the protection of personal data. Requests to access the datasets should be directed to Ingvar A. Bergdahl, ingvar.bergdahl@umu.se.

## Ethics Statement

The studies involving human participants were reviewed and approved by Ethical Review Board in Umea and the Florence Health Unit Local Ethical Committee. The patients/participants provided their written informed consent to participate in this study.

## Author Contributions

JK, TdK, SK, and JCK designed the research. IB, AJ, and DP organized the epidemiologic part of the work. TL performed the experiments, and JK analyzed the data. TdK and JCK supervised the study. JK, TdK, and JCK co-wrote and all authors commented on the paper.

## Conflict of Interest

The authors declare that the research was conducted in the absence of any commercial or financial relationships that could be construed as a potential conflict of interest.
