# Supplementary material for: Blood Transcriptome Response to Environmental Metal Exposure Reveals Potential Biological Processes Related to Alzheimer's Disease
Source: Front Public Health. 2020 Oct 21;8:557587. doi: 10.3389/fpubh.2020.557587 (PMC7609776; doi:10.3389/fpubh.2020.557587)
Supplement: Supplementary file 1 [file Data_Sheet_1.pdf]

Supplementary Figures S1

ppq plot of covariates included in the final models. The ppq plots visualize the distribution of p-values from a anova test when comparing the regression model with and without the respective covariate.

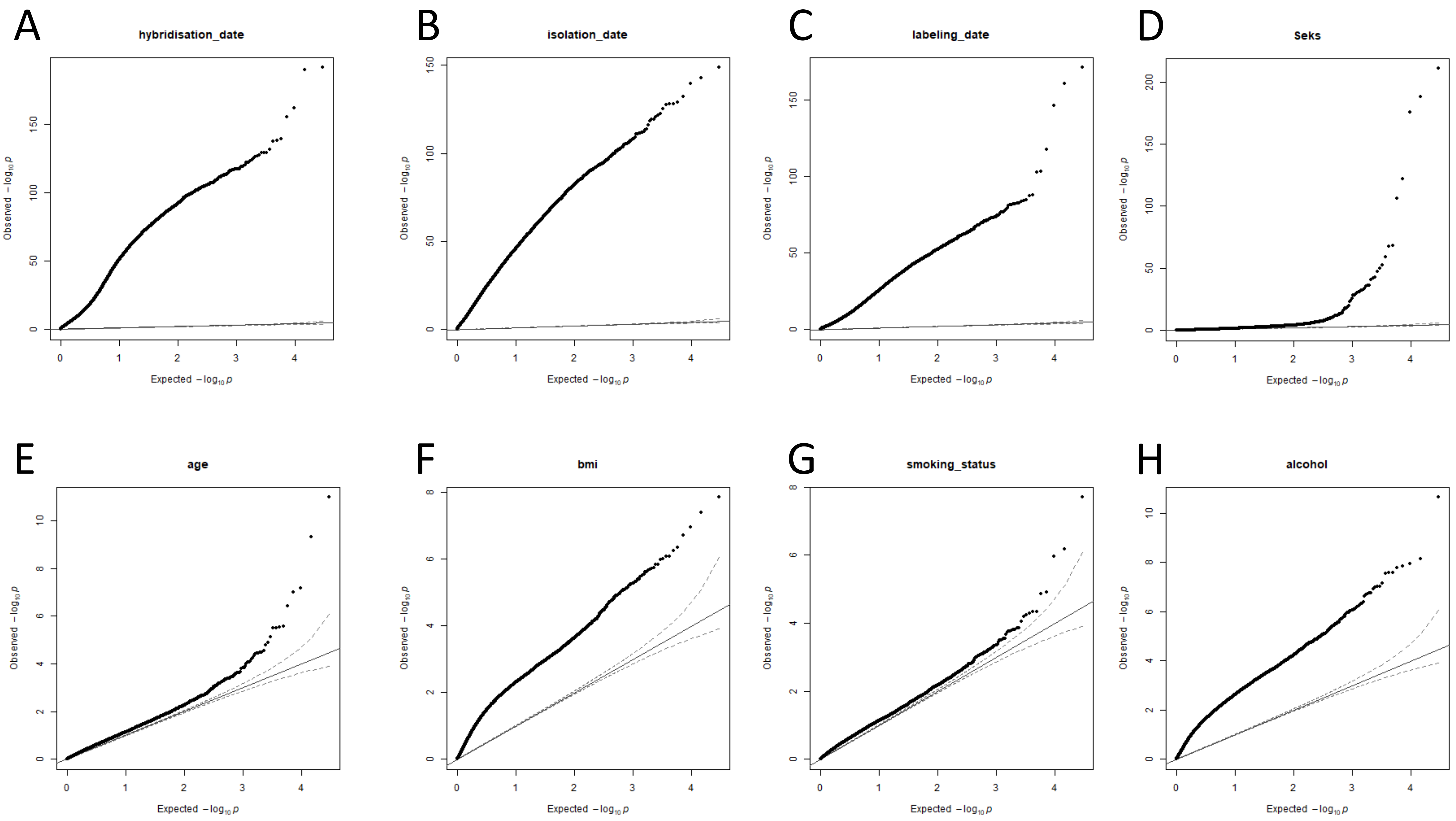

A
hybridisation_date
B
isolation_date
C
labeling_date
D
Seks
E
age
F
bmi
G
smoking_status
H
alcohol
Observed $-\log_{10} p$
Expected $-\log_{10} p$

Supplementary Figures S2

Overlap of Cd associated genes in males with two recent AD publication. The Venn diagramm show the overlap with the genetic meta-analysis of diagnosed AD patients by Kunkle *et al.,* 2019 (A) and the single cell transcriptomic analysis by Mathys *et al., 2019* (B).

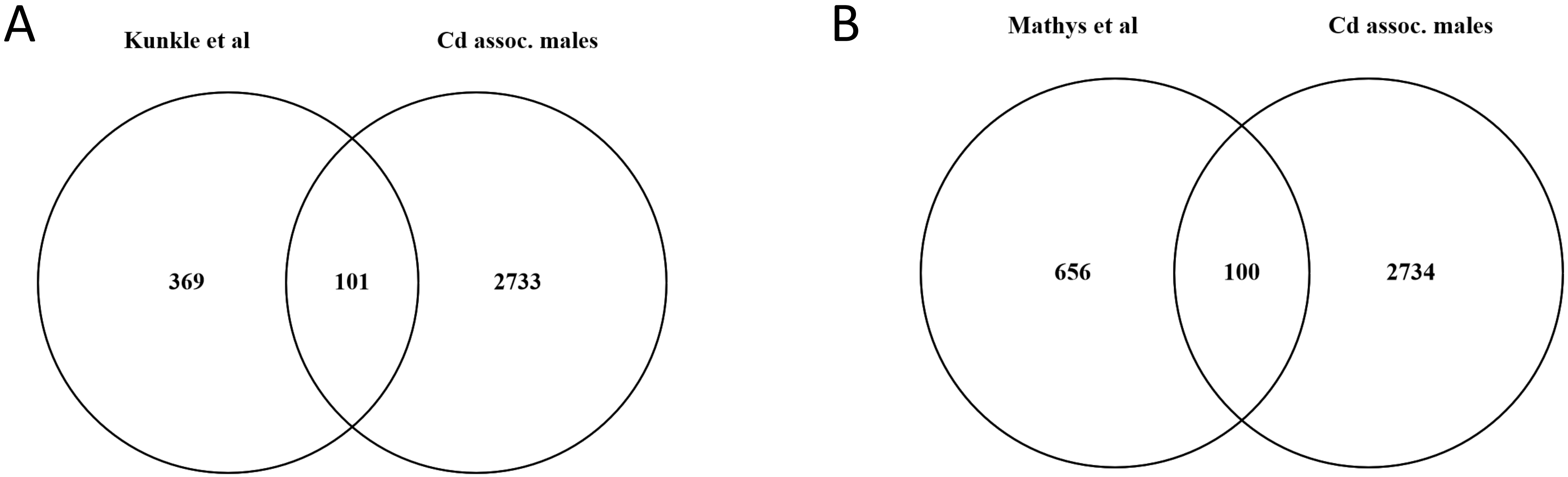
A
Kunkle et al
Cd assoc. males
369
101
2733
B
Mathys et al
Cd assoc. males
656
100
2734

Supplementary Figures S3

Expression of top 50 significant Cd associated genes in males across brain samples. The heatmap shows the expression in transcripts per million (TPM) for the top 50 genes ranked by significance across whole blood and multiple brain regions derived from the GTEx database (version 8) The data shows that the lage majority of genes identified in blood are similarly expressed across all brain tissue.

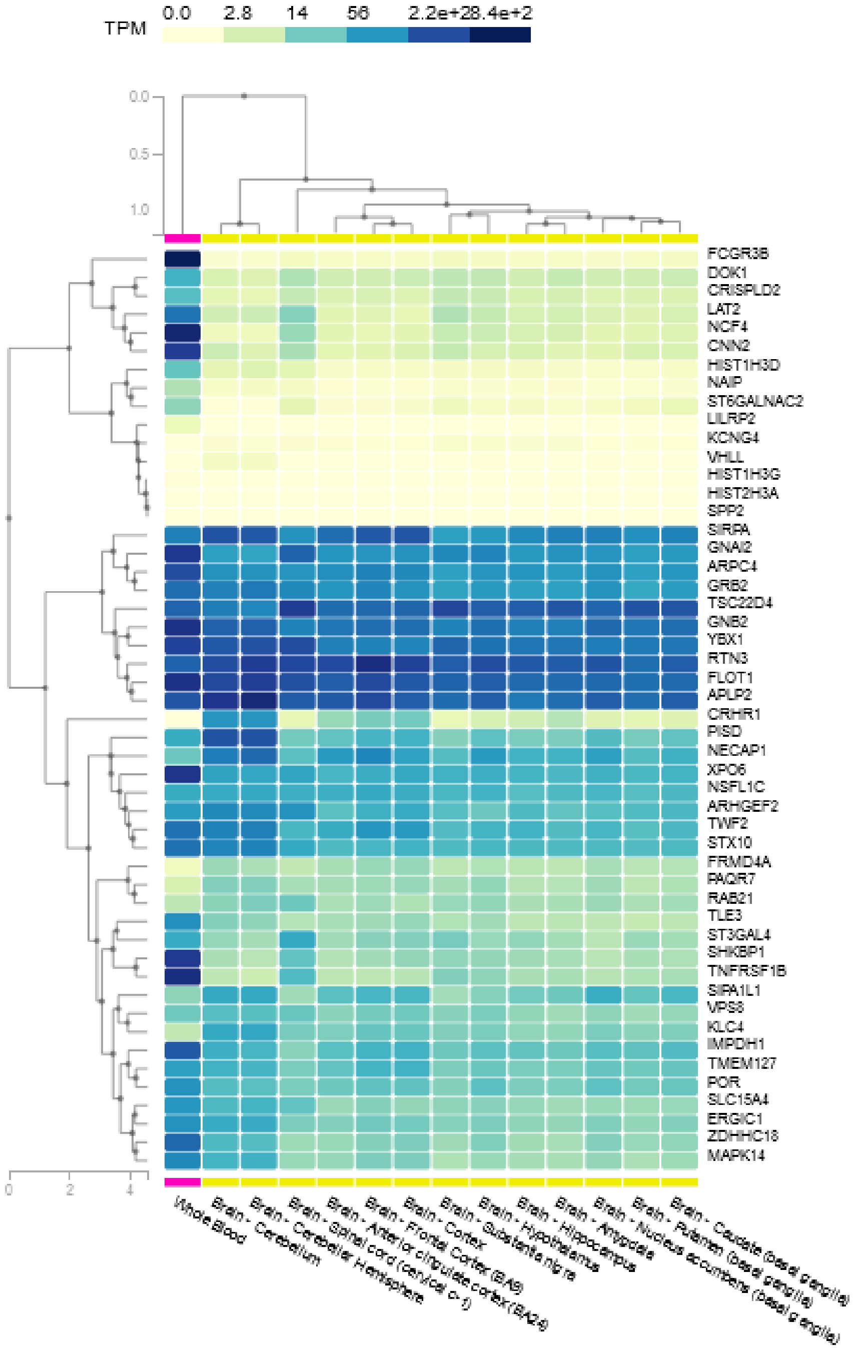
TPM
0.0
2.8
14
56
2.2e+2
8.4e+2
FCGR3B
DOK1
CRISPLD2
LAT2
NCF4
CNN2
HIST1H3D
NAIP
ST6GALNAC2
LILRP2
KCNG4
VHLL
HIST1H3G
HIST2H3A
SPP2
SIRPA
GNAI2
ARPC4
GRB2
TSC22D4
GNB2
YBX1
RTN3
FLOT1
APLP2
CRHR1
PISD
NECAP1
XPO6
NSFL1C
ARHGEF2
TWF2
STX10
FRMD4A
PAQR7
RAB21
TLE3
ST3GAL4
SHKBP1
TNFRSF1B
SIPA1L1
VPS8
KLC4
IMPDH1
TMEM127
POR
SLC15A4
ERGIC1
ZDHHC18
MAPK14
Whole Blood
Brain - Cerebellum
Brain - Cerebellar Hemisphere
Brain - Spinal cord (cervical c-1)
Brain - Anterior cingulate cortex (BA24)
Brain - Frontal Cortex (BA9)
Brain - Cortex
Brain - Substantia nigra
Brain - Hypothalamus
Brain - Hippocampus
Brain - Amygdala
Brain - Nucleus accumbens (basal ganglia)
Brain - Putamen (basal ganglia)
Brain - Caudate (basal ganglia)

Supplementary Figures S4

PPI network of Cd associated genes in males. The network was obtained from Reactome FIs using Cytoscape. To enable readability of this large network we applied MCL Cluster and only selected for clusters with a minimum of 40 connected nodes. This resulted in 8 subnetworks (A-H). Node sizes indicate the degree. Color indicates a calculated GeneScore derived from the multiplication of the β-coefficient and $-\log_{10}$(pvalue). A red shade indicates a significant positive, a blue shade a significant negative association of the respective transcript with Cd exposure in men.

A

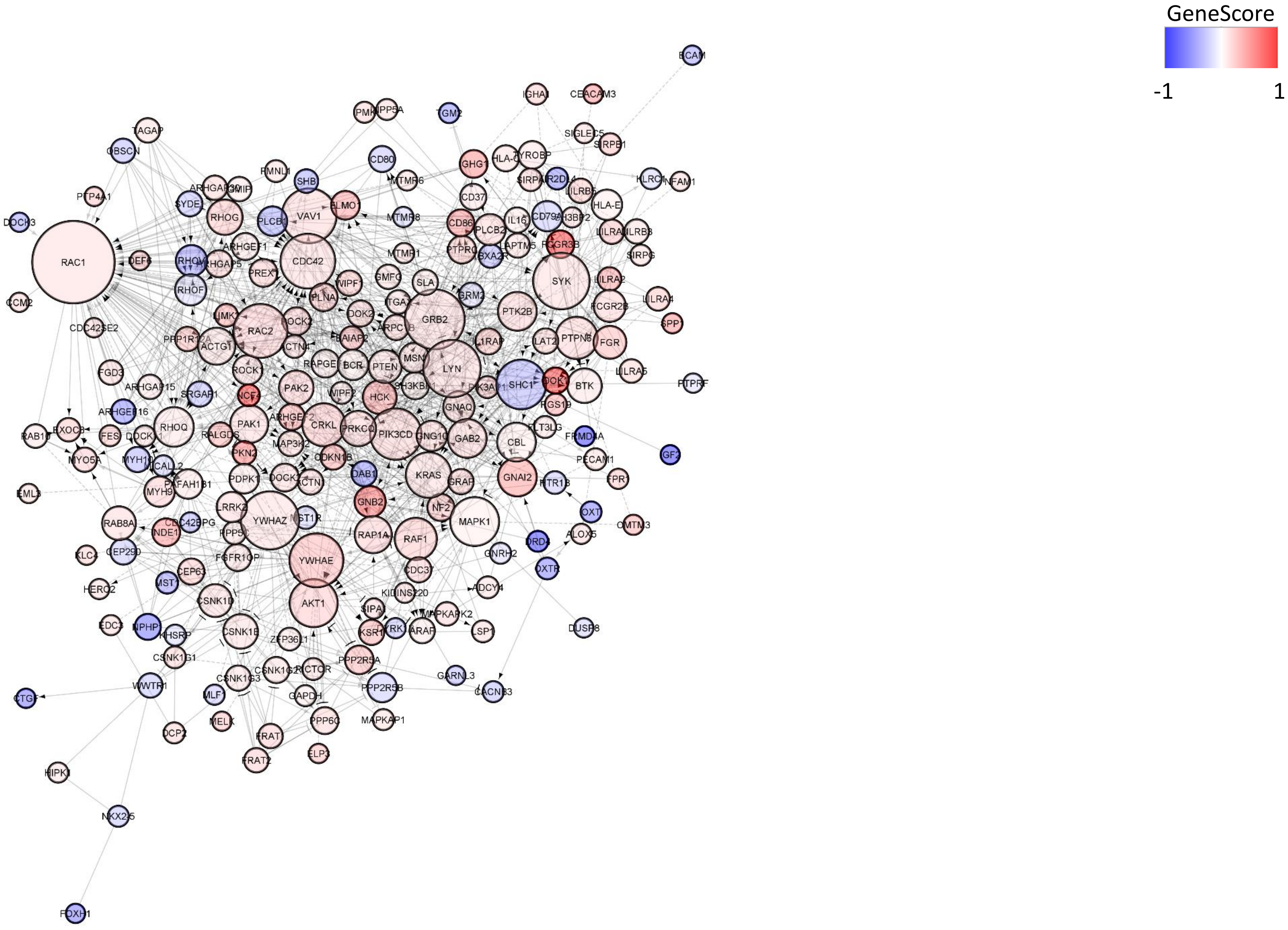
GeneScore
-1
1
RAC1
CDC42
VAV1
RAC2
GRB2
LYN
SYK
SHC1
PIK3CD
KRAS
MAPK1
YWHAZ
YWHAE
AKT1
RAF1
RAP1A
GNAI2
CBL
BTK
PTK2B
HCK
CRKL
PAK1
PAK2
RHOQ
RHOG
RHOF
GAB2
FGR

B

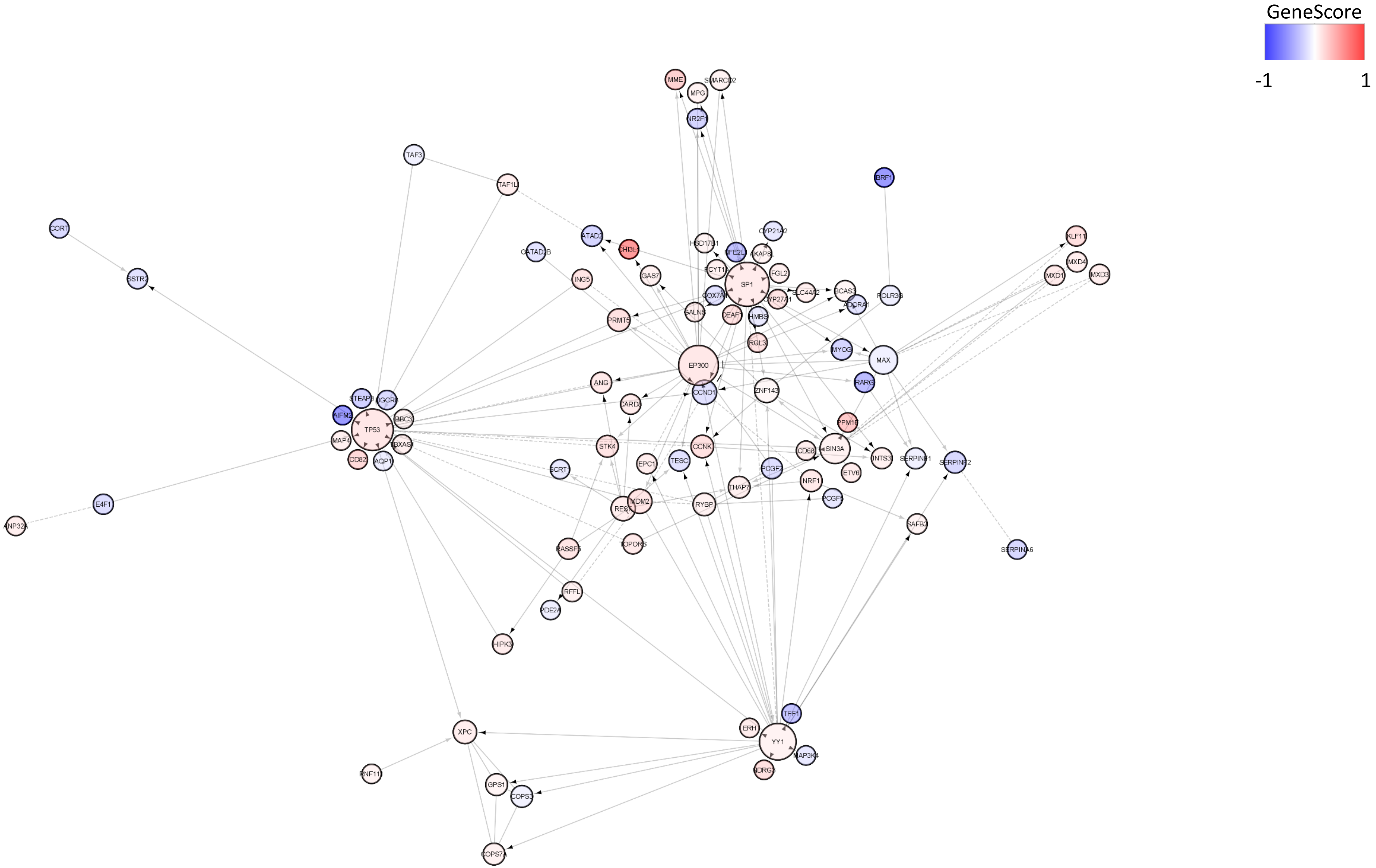

GeneScore
-1
1
MME
MPG
NR2F1
TAF3
TAF1L
BRF1
CORT
SSTR2
ATAD2
GATAD2B
ING5
GAS7
SP1
FGL2
HMBS
PRMT5
DEAF1
RGL3
MYOG
MAX
RARG
EP300
CCND1
ZNF143
ANG
CARD8
STEAP3
DGCR8
AIFM2
TP53
BBC3
MAP4
CD82
AQP1
E4F1
ANP32A
KLF11
MXD4
MXD1
MXD3
PPM1D
SIN3A
CD68
INTS3
ETV6
SERPINF1
SERPINF2
SERPINA6
SAFB2
STK4
CCNK
EPC1
TESC
PCGF2
THAP7
NRF1
PCGF3
RYBP
MDM2
TOPORS
RASSF5
RFFL
PDE2A
HIPK3
XPC
GPS1
COPS3
RNF11
ERH
YY1
TFF1
NDRG3

C

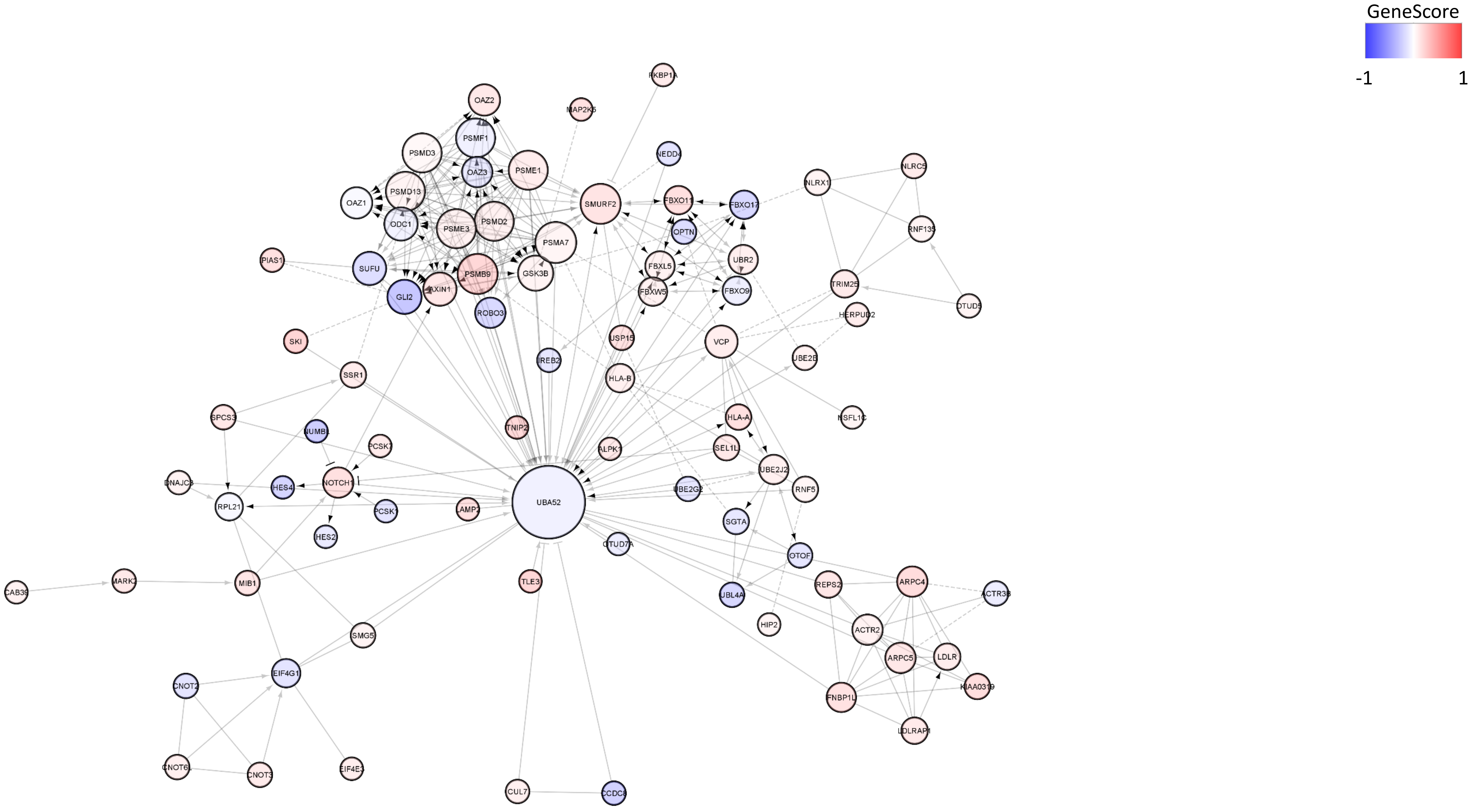
GeneScore
-1
1
OAZ2
PSMF1
PSMD3
OAZ3
PSME1
PSMD13
OAZ1
ODC1
PSME3
PSMD2
PSMA7
SMURF2
FBXO11
FBXO17
OPTN
UBR2
FBXL5
FBXW5
FBXO9
PIAS1
SUFU
PSMB9
GSK3B
AXIN1
GLI2
ROBO3
SKI
SSR1
SPCS3
NUMBL
PCSK7
NOTCH1
HES4
DNAJC3
RPL21
PCSK1
HES2
AMP2
UBA52
TNIP2
ALPK1
IREB2
USP15
HLA-B
VCP
HLA-A
SEL1L
UBE2J2
UBE2G2
SGTA
OTUD7A
OTOF
RNF5
UBL4A
HIP2
TLE3
MIB1
MARK2
CAB39
SMG5
EIF4G1
CNOT2
CNOT6L
CNOT3
EIF4E3
CUL7
CCDC8
NEDD4
MAP2K5
FKBP1A
NLRX1
NLRC5
RNF135
TRIM25
OTUD5
HERPUD2
UBE2E
NSFL1C
REPS2
ARPC4
ACTR3B
ACTR2
ARPC5
LDLR
KIAA0319
FNBP1L
LDLRAP1

D

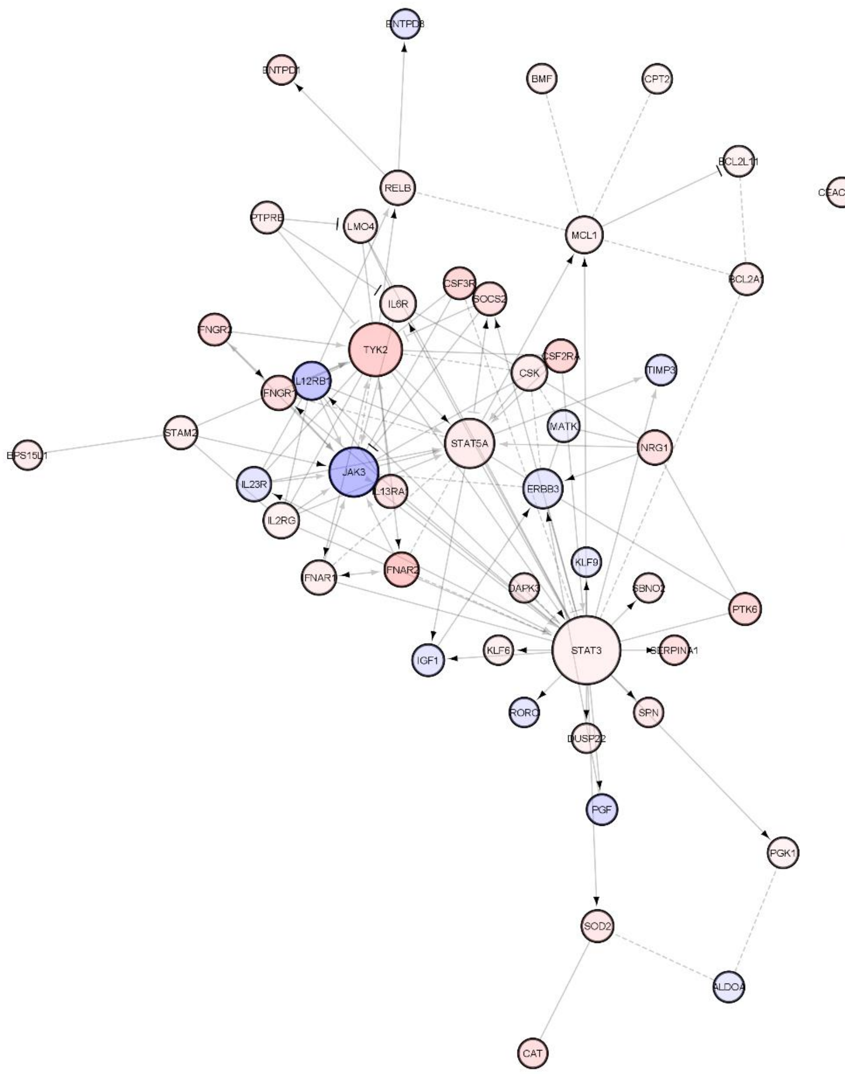

ENTPD8
ENTPD1
BMF
CPT2
BCL2L11
RELB
PTPRE
LMO4
MCL1
BCL2A1
IL6R
CSF3R
SOCS2
TYK2
CSK
CSF2RA
TIMP3
STAT5A
MATK
NRG1
JAK3
ERBB3
STAM2
EPS15L1
IL23R
IL2RG
KLF9
DAPK3
SBNO2
PTK6
STAT3
KLF6
IGF1
SERPINA1
SPN
DUSP22
PGF
PGK1
SOD2
ALDOA
CAT

E

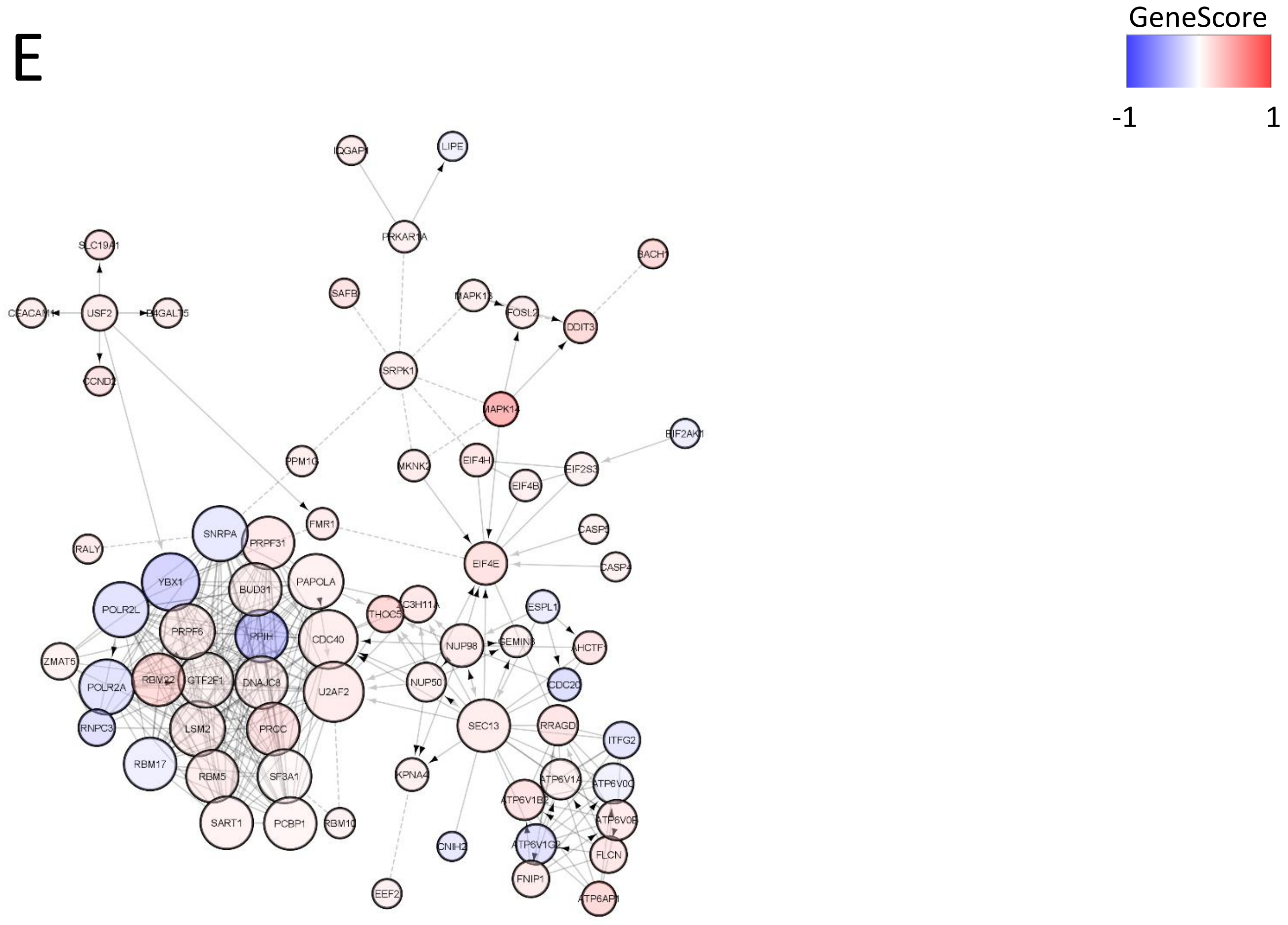

GeneScore
-1
1
LIPE
PRKAR1A
SLC19A1
USF2
CEACAM1
CCND2
SAFB
MAPK13
FOSL2
DDIT3
SRPK1
MAPK14
PPM1G
MKNK2
EIF4H
EIF4B
EIF2S3
EIF2AK1
FMR1
RALY
SNRPA
PRPF31
YBX1
BUD31
PAPOLA
EIF4E
CASP4
POLR2L
PRPF6
PPIH
CDC40
THOC5
ZC3H11A
ESPL1
NUP98
GEMIN8
AHCTF1
ZMAT5
RBM22
GTF2F1
DNAJC8
U2AF2
NUP50
CDC20
POLR2A
RNPC3
LSM2
PRCC
SEC13
RRAGD
ITFG2
RBM17
RBM5
SF3A1
KPNA4
ATP6V1A
ATP6V0C
SART1
PCBP1
RBM10
CNIH2
FLCN
FNIP1
ATP6AP1
EEF2

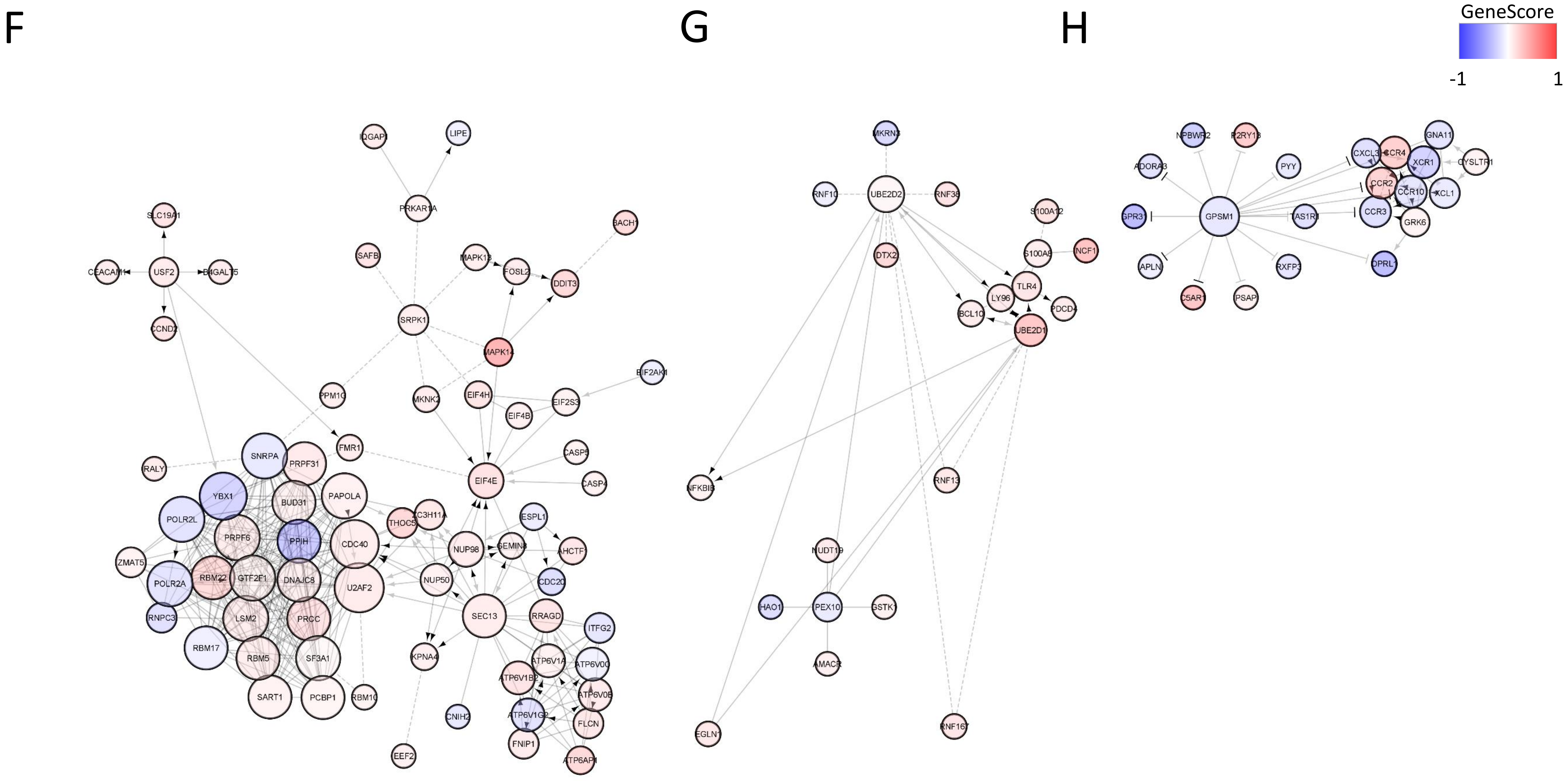
F
G
H
GeneScore
-1
1
IQGAP1
LIPE
PRKAR1A
SLC19A1
BACH1
CEACAM1
USF2
B4GALT5
SAFB
MAPK13
FOSL2
DDIT3
CCND2
SRPK1
MAPK14
EIF2AK1
PPM1G
MKNK2
EIF4H
EIF4B
EIF2S3
FMR1
CASP5
RALY
SNRPA
PRPF31
EIF4E
CASP4
YBX1
PAPOLA
BUD31
ZC3H11A
THOC5
POLR2L
ESPL1
PRPF6
PPIH
CDC40
NUP98
GEMIN8
AHCTF1
ZMAT5
RBM22
GTF2F1
DNAJC8
POLR2A
U2AF2
NUP50
CDC20
RNPC3
LSM2
PRCC
SEC13
RRAGD
ITFG2
RBM17
RBM5
SF3A1
KPNA4
ATP6V1A
ATP6V0C
ATP6V1B2
ATP6V0B
SART1
PCBP1
RBM10
CNIH2
ATP6V1G2
FLCN
FNIP1
EEF2
ATP6AP1
MKRN3
RNF10
UBE2D2
RNF38
S100A12
DTX2
S100A8
NCF1
TLR4
LY96
BCL10
PDCD4
UBE2D1
NFKBIB
RNF13
NUDT19
HAO1
PEX10
GSTK1
AMACR
EGLN1
RNF167
NPBWR2
P2RY13
GNA11
CXCL3
CCR4
XCR1
ADORA3
PYY
CYSLTR1
CCR2
CCR10
XCL1
GPR31
GPSM1
TAS1R1
CCR3
GRK6
APLN
RXFP3
OPRL1
C5AR1
PSAP
